# The promise and peril of AI for disability health equity: A justice-centered narrative review

**DOI:** 10.1371/journal.pdig.0001663

**Published:** 2026-09-01

**Authors:** Jyoti Dalal, Douglas Teodoro, Minerva Rivas Velarde

**Affiliations:** 1 Institute of Global Health, Faculty of Medicine, University of Geneva, Geneva, Switzerland; 2 Department of Radiology and Medical Informatics, University of Geneva, Geneva, Switzerland; 3 HES-SO University of Applied Arts Sciences and Arts of Western Switzerland, Geneva, Switzerland; 4 The Institute for Ethics, History, and the Humanities (iEH2), Faculty of Medicine, University of Geneva, Switzerland; 5 Global Health Centre, The Graduate Institute (IHEID), Geneva, Switzerland; National Tsing Hua University, TAIWAN

## Abstract

Despite the promising potential of Artificial Intelligence (AI) models to enhance health equities for persons with disabilities, poorly designed applications risk exacerbating inequities. To address this, we conducted a narrative review using disability justice frameworks as an analytical lens to evaluate how AI applications operationalize disability and health equity across various domains. AI models demonstrate potential in early intervention, personalized care, equitable resource allocation, assistive technologies, and health surveillance. However, most AI models rely primarily on biomedical and functional data, often trained on biased datasets that neglect social and structural determinants of disability. As a result, these applications insufficiently capture broader dimensions of wellbeing, including capabilities, recognition, and structural justice, limiting their effectiveness in addressing health inequities. To advance health equity for persons with disabilities, AI applications must incorporate disability-inclusive datasets, participatory co-design with persons with disabilities, auditing for fairness, and real-world validation. Integrating clinical, social, and structural dimensions in a justice-oriented framework can help AI models move beyond narrow biomedical models toward more inclusive, context-sensitive, and equitable health systems.

## 1. Introduction

Despite the promising potential of Artificial Intelligence (AI) models to enhance health equities for persons with disabilities, they could cause more harm if not carefully designed. In this work, we aim to understand this better by taking a justice-centered approach. We conducted a narrative analysis by extracting the available evidence integrating disability justice theories with a critical review of machine learning applications across various domains. We also report the limitations of machine learning models along with giving recommendations for advancing these models to better help in enhancing disability health equity.

Full and equal participation in all aspects of life is a fundamental human right, protected in international frameworks such as the United Nations Convention on the Rights of Persons with Disabilities [1]. The CRPD defines persons with disabilities (PwDs) as those with “*long-term physical, mental, intellectual or sensory impairments which, in interaction with various barriers, may hinder their full and effective participation in society on an equal basis with others.*” This socially grounded definition emphasizes that disability arises not solely from individual impairments, but from their interaction with environmental, social, and institutional barriers. Despite this progressive and inclusive vision, disabled communities continue to face widespread social exclusion, which directly contributes to deep health inequities. These disparities are not inherent to impairments but stem from systemic neglect, ableist norms, and institutional barriers, making the pursuit of health equity fundamentally a matter of social justice.

Traditional approaches to equity often focus narrowly on access to healthcare services, overlooking deeper structural and relational injustices. Disabled populations do encounter physical barriers, discrimination, lack of accommodations, exclusion from research, and stigmatizing attitudes among healthcare providers [2,3]. Iris Marion Young’s concept of structural injustice highlights how social institutions and norms perpetuate marginalization, while Nancy Fraser’s three-dimensional framework of redistribution, recognition, and representation underscores the need for material support, cultural affirmation, and inclusive decision-making to dismantle multidimensional inequities [3–6].

The capability approach, developed by Sen and Nussbaum, emphasizes individuals’ real freedoms (or capabilities) and their actual ability to function and flourish [7,8]. Nussbaum’s central capabilities: bodily health, bodily integrity, affiliation, and control over one’s environment, require not only barrier removal but proactive support such as assistive technologies, community-based care, and autonomy-enhancing services. Tom Shakespeare’s relational model bridges medical and social perspectives, advocating for individualized, dignity-centered care that respects diversity without reinforcing dependency [6]. Paul Farmer’s concept of structural violence situates these inequities within global systems of poverty, exclusionary policies, and under-resourced services, framing health equity as a human rights obligation rather than a charitable provision [9]. Collectively, these frameworks demonstrate that advancing health equity requires transforming systems not originally designed for disabled lives, addressing both embodied experiences of impairment and the social structures that marginalize.

These justice frameworks differ in emphasis, whether on structural injustice, recognition, lived capabilities, relational care, or global inequities, but converge in showing that health systems must be transformed to support disabled lives. Crucially, the challenge is not only theoretical but practical: how can we empirically assess whether these frameworks are reflected in real-world outcomes? Here, emerging computational tools such as Machine Learning (ML) and AI open possibilities to interrogate disability inequities with new depth and scale. For example, the capability approach already draws on mathematical and economic reasoning, and machine learning can extend this by testing whether existing datasets adequately capture core freedoms such as bodily integrity and affiliation, or whether prevailing health metrics obscure recognition and representation dimensions. Similarly, predictive models and natural language processing could help in identifying patterns of exclusion, reveal biases in provider practices or medical records, and surface marginalized voices often missing from policy debates. When used critically and ethically, these approaches allow us to ask not only “what outcomes exist,” but also “are we collecting the right indicators, and asking the right questions?”

To address these gaps, we adopt a **justice-oriented analytical lens** drawing on key dimensions from the disability justice frameworks - capabilities, redistribution, recognition, and representation, and structural inequities, to evaluate how AI applications operationalize disability health equity in practice. However, translating theory into practice is challenged by gaps in reliable disability data. The CRPD’s broad, socially grounded definition puts forward a challenge to document barriers as well as the well-being of individuals. Machine learning offers tools to analyze complex, fragmented disability datasets, uncover hidden patterns, and produce dynamic classifications beyond traditional statistical methods [10,11].

## 2. Methods: Narrative review approach

We conducted a purposeful, concept-driven search in databases like PubMed and Google Scholar using keywords related to disability (“impairment”, “disability”, “persons with disabilities”, “functioning”, “ICF”), AI/ML (“artificial intelligence”, “machine learning”, “deep learning”, “natural language processing”), and health equity (“health disparities for persons with disabilities”, “justice”, “AI fairness”, “AI ethics”, “disability health equity”).

**Inclusion criteria**: We included (1) empirical studies applying AI/ML in health or disability-related contexts, (2) studies addressing equity, bias, or social determinants of health, and (3) foundational theoretical works relevant to disability justice and health equity (e.g., capabilities lens, structural injustice), published between 2000 and 2025.

**Exclusion criteria:** Studies focused solely on technical AI/ML development without relevance to disability or health equity were excluded.

Given the interdisciplinary and evolving nature of the field, and the relatively limited and heterogeneous evidence base, a narrative review was used rather than a systematic review. The current literature is characterized by conceptual diversity, emerging empirical contributions, and varying methodological quality, which limits the value of a formal systematic review. Instead, a narrative approach allows for a more flexible and critical analysis of how the field is evolving. Studies were therefore purposefully selected for their conceptual and empirical contributions, organized thematically, and analyzed through disability justice frameworks.

**Bias mitigation**: The first author conducted the initial identification and drafting, which co-authors reviewed and validated. Iterative discussions refined themes and ensured balanced representation.

## 3. Background: Disability, health inequities, and data-driven approaches

### 3.1. Health inequities among PwDs

The urgency of integrating justice frameworks with data-driven approaches becomes evident when considering healthcare, where systemic inequities for PwDs are particularly stark. Globally, 1.3 billion people (around 16% of the world’s population) live with a significant disability; PwDs do have poorer health outcomes as compared to the general population [12]. These disparities are not inherent to impairment itself but are shaped by structural barriers, including inaccessible facilities and equipment, negative provider attitudes, inadequate training, communication barriers, and unaffordable care [13,14]. PwDs are also less likely to receive preventive services, screening recommendations, or continuity of care [12].

The 2025 Global Disability Inclusion Report underscores that robust, disaggregated data systems are essential for unveiling such inequities and shaping effective responses [15]. It calls for:

- Aligning health policies with the CRPD,
- designing health systems around accessibility, inclusive infrastructure, and assistive technologies,
- training providers for person-centered, disability-inclusive care, and
- involving Organizations of PwDs in planning and decision-making.

Taken together, these insights highlight that advancing health equity for PwDs is inseparable from advancing disability data equity. Without accurate, inclusive, and justice-informed data collection, health inequities remain obscured and interventions risk being misdirected. This makes the design and use of disability datasets central to the pursuit of inclusive health systems.

### 3.2. Data-driven approach and its promises

Disability datasets are frequently marked by heterogeneity, idiosyncrasies, and incompleteness, which collectively hinder the development of robust and accurate statistical models [16,17]. *Heterogeneity* arises from differences in how disability is defined and measured across datasets. For instance, surveys like the World Health Survey use self-reported functional limitations, while administrative datasets (e.g., social care records) may define disability in terms of service eligibility or diagnostic categories [17,18]. *Idiosyncrasies* could be seen in inconsistent coding practices, such as combining intellectual and developmental disabilities into a single category in one dataset while separating them in another, or in the use of vague categories like “other” that lack interpretive clarity [19]. *Incompleteness* is also common, some health records exclude disability status altogether, while large-scale datasets may underrepresent people living in institutions, group homes, or those with complex communication needs, leading to systematic gaps [16,18]. These limitations not only reduce statistical power but can bias estimates, obscure intersectional disparities, and weaken the evidence base needed for inclusive health and social policy [16,17].

These challenges are particularly pronounced in low- and middle-income countries (LMICs), where infrastructure and political will for collecting disability-disaggregated data are limited. Fewer than 50 countries globally included such data in national surveys [2], and analyses of 1,297 datasets from 133 LMICs (2009–2018) found that fewer than a third captured disability-related questions [20]. Only half of countries and 15% of datasets contained functional difficulty questions meeting international standards [21], 68 countries included the Washington Group Short Set, and data on education, health, living standards, and wellbeing were available from just 35 countries [22].

These limitations compromise the reliability and generalizability of analyses and models, reducing their utility for policymaking. Nonetheless, underutilized imperfect data still offers valuable insights for targeted interventions, tracking inequities, and guiding resource allocation. For example, the World Health Survey and Washington Group Short Set, despite underrepresenting certain disabilities and limited context, have effectively highlighted disparities in healthcare access, education, and employment outcomes [16,23]. Leveraging available data transparently supports iterative learning and more responsive policy design.

Machine learning offers the potential to work productively with imperfect disability datasets [24]. By handling unstructured, multidimensional, and incomplete data, it can detect non-linear relationships and hidden patterns across variables such as disability type, healthcare quality, education, and employment [25–27]. However, its benefits are conditional: without inclusive datasets, governance, and system readiness, AI may amplify inequities. For example, AI chatbots have already produced biased outputs, highlighting the need for clearer guidelines to protect marginalized groups [28]. Ultimately, whether AI strengthens equity depends on structural factors including data quality, care pathways, and infrastructure [29,30].

## 4. Applications of AI/ML in the disability contexts

In this section, we explore concrete areas in which AI/ML models could enhance disability data analysis and help move the field toward more inclusive evidence-based decision-making.

### 4.1 Personalized care and early intervention

ML increasingly supports personalized care for PwDs by analyzing diverse data sources such as Electronic Health Records (EHR), wearables, and individual profiles, including social determinants of health, to identify risk patterns, forecast functional decline, and guide tailored interventions. Predictive models have been used to forecast long-term disability and mobility limitations in older adults, supporting early planning and care coordination [31–33]. Equity-oriented modeling is emerging, with approaches addressing bias in predicting hospital length of stay for individuals with learning disabilities [34]. In rehabilitation, deep learning pipelines such as PrimSeq classify functional movements during stroke recovery [35], while platforms like Walk4Me use smartphone sensors for remote mobility monitoring [36]. From a justice perspective, these models partially align with a capabilities lens by addressing bodily health [7,8], but they often overlook autonomy, social participation, and environmental barriers.

### 4.2 Equitable resource allocation

Unequal access to specialized disability services, especially in rural and underserved areas, remains a critical challenge. ML could help address this gap by analyzing multi-modal data, such as geographic, demographic, insurance claims, and social determinants, to predict regions with the highest needs for assistive devices, home care, and accessible infrastructure, facilitating more equitable distribution of resources [37]. Predictive analytics has demonstrated potential to improve rehabilitation service delivery and outcomes under policy expansions [38,39]. Geospatial tools such as the AI-driven Accessibility Mapping Tool further support resource planning [40]. These models address recognition and redistribution perspectives [5] but insufficiently capture structural inequities and representation of diverse disability experiences [3,9].

### 4.3 Identifying barriers and facilitators to access

ML techniques can also play a role in identifying the social determinants of health that affect PwDs. Analyzing transportation data, housing records, and patient feedback could help in identifying structural patterns influencing access to care. Natural Language Processing (NLP) offers tools to work with unstructured clinical text found in EHR, where information on patient functioning, accessibility issues, or caregiver arrangements is sometimes recorded but not captured in structured fields. Studies have extracted functioning-related information from EHR to align with frameworks like the International Classification of Functioning, Disability and Health [41]. Others have identified mobility limitations using keyword-based approaches [42] or extracted mental functioning data for disability determination [43]. Additionally, multilingual NLP research demonstrates feasibility across diverse linguistic contexts, including Brazilian Portuguese and French [44,45]. NLP applications enhance recognition by extracting functioning data from unstructured text, but reliance on clinical narratives may reinforce misrecognition if lived experiences are underrepresented, highlighting gaps in justice-oriented frameworks [3,5].

### 4.4 Better health monitoring and surveillance

ML models have the potential to address underrepresentation of PwDs in conventional surveillance systems [2] by analyzing additional data sources, such as wearables, insurance claims, and telemedicine platforms. For example, during the COVID-19 pandemic, ML-driven surveillance identified elevated mortality rates among institutionalized PwDs, informing targeted outreach and vaccination efforts [46]. While these systems could help reduce structural inequities [3,9], insufficient representation of PwDs in datasets risks reinforcing existing disparities [2,46].

### 4.5 Assistive technologies

Lastly, ML has significantly advanced assistive technologies that enhance autonomy and daily functioning for PwDs. Recent advances integrate machine learning into devices that support mobility, communication, and environmental awareness [47]. For example, ML-driven wheelchair systems using muscle signals or eye movements have demonstrated high accuracy in gesture recognition and gaze tracking [48,49]. Wearable devices for visually impaired users improve real-time obstacle detection and spatial navigation [50]. AI-powered communication tools, including sign-to-speech translation and activity recognition systems, further enhance independence [51]. Assistive technologies do support capabilities and autonomy [7,8] but may exclude some users without participatory design, highlighting the need for representation in development.

## 5. Evaluating AI/ML applications through justice frameworks

To address whether AI/ML applications reflect the justice-oriented frameworks, we apply key dimensions derived from disability justice theories: real freedoms, redistribution, recognition, representation perspectives, structural inequities lens, as well as relational care perspectives [4–9]. Across the reviewed applications, most models primarily operationalize disability through biomedical or functional indicators (e.g., mobility, ADL limitations), capturing only some aspects of capabilities and relational wellbeing [31–33,52]. For instance, predictive models of functional decline capture aspects of bodily health but rarely account for social participation, autonomy, or environmental barriers, partially reflecting a capabilities lens [7,8].

NLP-based extraction often relies on clinical descriptors, limiting recognition and representation of lived experience [41–43]. Few models address structural inequities, such as barriers in access to care or socio-economic exclusion, central to justice frameworks [3,5,9]. Overall, AI/ML applications reflect narrow operationalizations of disability, leaving social and structural determinants underrepresented [53].

## 6. Limitations and challenges of the current AI/ML approaches

While ML holds significant promise for supporting the advancement of care, planning, and technology related to disability, critical methodological and conceptual challenges need to be addressed to ensure that future research is equitable, effective, and inclusive.

One major limitation is the lack of *sufficiently detailed and unbiased disability* data. Many studies rely on datasets created for broader purposes, where disability is included only as a secondary measure or captured through proxy indicators (e.g., wheelchair-related keywords) rather than verified diagnoses [31–33,42]. These datasets often underrepresent PwDs in public health surveillance systems and EHR [2,46], limiting the ability of models to reflect complex social, cultural, and structural dimensions of disability. Equity-aware approaches, such as reweighting underrepresented populations and incorporating fairness constraints, remain rare [34].

Closely related are challenges of *generalizability and real-world applicability*. ML models may perform well in controlled settings but often have limitations when transferred across care contexts, age groups, or disability types [35,36,48–50]. Technologies like wearables or gesture-based systems frequently assume stable signal quality or require individualized calibration. Similarly, while multilingual NLP is technically feasible [44,45], models rarely account for language or cultural differences in describing disability, reducing reliability in practice.

Beyond technical concerns, there are *deep conceptual and philosophical ambiguities* in how disability is defined and operationalized. Different models: medical, social, biopsychosocial, or rights-based, offer distinct interpretations of disability, impairment, and participation [3]. Many applications adopt a purely medical perspective, which shapes data collection (e.g., diagnoses vs. social barriers), outcome definitions (e.g., physical improvement vs. independence), and criteria for “success.” For example, a rehabilitation algorithm may define success as regaining walking ability while overlooking whether an individual can return to work, school, or community life. Although the WHO’s International Classification of Functioning, Disability and Health provides a comprehensive framework integrating health conditions with personal and environmental factors [54], few ML studies explicitly reference it. As a result, conceptual and definitional biases are embedded in AI systems, influencing not only model outputs but also broader societal narratives of disability in technology and healthcare.

*Scope and interpretability* of disability-focused ML remain narrow. NLP studies often emphasize mobility or cognition while underexploring communication, pain, fatigue, or environmental factors [43]. Limited annotated datasets, variability in clinical language, and lack of context sensitivity further constrain model performance. Few models incorporate intersectional social determinants such as housing, education, or race, which shape disability experiences. Even technically sophisticated tools, including PrimSeq [35] or CNN-based interfaces, are often difficult to interpret or scale, with limited real-world integration, usability testing, or long-term impact.

These limitations highlight the urgent need for inclusive design, participatory co-development with PwDs, and real-world validation to ensure that ML applications are equitable, contextually relevant, and capable of addressing structural and social dimensions of disability.

## 7. Ethical considerations

Applying ML in disability health contexts raises significant ethical concerns that require careful attention to prevent harm and uphold disability rights. These concerns are not generic AI ethics issues: disability-related data is uniquely sensitive, socially embedded, and connected to systemic inequities. In line with the principles of the Declaration of Helsinki [55], additional efforts are required to ensure that socially disadvantaged populations, including PwDs, are appropriately included in research and not systematically excluded from its benefits.

### 7.1. Privacy and data security

Disability status, functional assessments, and assistive technology use are deeply sensitive. Misuse can expose PwDs to discrimination in employment, education, or insurance [56]. Robust encryption, federated learning, and strict compliance with GDPR and the EU AI Act are essential [57]. Special safeguards are also needed for disability registries, clinical rehabilitation records, and unstructured notes containing identifiable narratives about functioning.

### 7.2. Informed consent and data governance

Standard consent models rarely account for how disability data is collected (e.g., via caregivers, social services, or family) or reused in secondary research or if they were excluded due to a lack of accessibility practices. PwDs often have limited control over how their lived experiences are codified in machine learning datasets [58]. Rivas Velarde et al. suggest framing consent as a compositional act, explicitly defining the data subject (or proxy), specific use, and receiving agent(s). This enhances transparency, accountability, and trust, allowing participants to adjust or revoke consent over time [59]. Embedding choice complements disability-positive training data and evolving disability culture [60].

### 7.3. Equity of access

AI solutions built without accessibility considerations risk reproducing inequities. ML-driven health platforms may assume visual, auditory, or cognitive abilities that exclude some PwDs. Connectivity and cost barriers disproportionately affect people with disabilities in low-resource contexts. Inclusive design should be paired with disability-inclusive benchmarks and metrics [61], ensuring access and usability are evaluated against socially grounded definitions of disability, not only technical criteria.

### 7.4. Justice and fairness

Technical safeguards alone are insufficient. AI should complement not replace the expertise of clinicians, caregivers, and PwDs themselves. Systems overemphasizing medicalised outcomes may devalue autonomy, participation, or lived experience. Fairness evaluation should go beyond clinical accuracy to assess whether outcomes align with holistic goals such as independence, participation, and accessibility. Involving PwDs in co-design and evaluation ensures technologies reflect disability-positive values, preventing harms such as misrecognition, stereotyping, or dehumanization [60,62].

Viewing privacy, consent, access, and fairness through this lens transforms abstract principles into concrete guidance for the design, governance, and deployment of AI in disability health. Embedding these considerations allows data-driven approaches not only to prevent harm but actively to advance health equity for PwDs.

## 8. Recommendations for advancing AI/ML applications in the disability contexts

To ensure that AI/ML models contribute equitably to the lives of PwDs, we synthesize key technical and design priorities into a justice-oriented framework emphasizing participation, contextual sensitivity, and structural equity. These priorities build on literature underscoring the need of collecting disability-inclusive datasets that capture variation in functioning, environments, and care needs beyond merely biomedical data [3,42,43,54], alongside participatory approaches involving PwDs in data collection, annotation, and evaluation [60,61]. At the model level, concerns around fairness and generalizability highlight the importance of evaluating performance across diverse populations and real-world contexts, accounting for variation across disability types, gender, race, and geography [31–36,49,53]. Advances in natural language processing further point to expanding beyond clinical domains to include social participation, communication, and environmental barriers, particularly within fragmented health and social care data [41,43,63,64]. Increasing emphasis on model interpretability reinforces the need for transparency, trust, and alignment with user needs in real-world deployment [33,65,66].

Table 1 translates these priorities into guiding questions across the AI/ML lifecycle, ranging from the data collection to deployment, emphasizing their interdependence rather than treating them as separate challenges. It highlights how choices about data, outcomes, and evaluation shape whose experiences are recognized and whose needs are addressed. Across domains, the meaningful involvement of PwDs emerges as a cross-cutting requirement, positioning them as active contributors in defining categories, evaluation metrics, and system objectives. By linking each recommendation to the principles of recognition, real freedoms, and structural equity, the framework illustrates how AI/ML systems can reproduce or mitigate inequalities and provides a practical tool for embedding justice considerations into development and implementation.

**Table 1:** Recommendations, guiding questions, and their connection with the justice-theories.

Recommendations	Guiding questions	Connection with the justice theories
1. Disability-inclusive datasets	Do datasets reflect diverse disability experiences, environments, and identities? Are PwDs involved in data collection, annotation, and validation? Do datasets capture social and environmental factors beyond medical data?	Supports recognition and representation; enables multidimensional capabilities.
2. Algorithmic fairness	• Are outcomes equitable across disability type, gender, race, and geography? • How is fairness defined, and does it reflect lived experiences? • Are PwDs involved in defining evaluation metrics and success criteria?	Mitigates structural injustice; promotes equitable outcomes across groups.
3. Generalizability	• Has the model been validated in diverse, real-world contexts? • Does performance vary across underrepresented populations? • Are adaptive or personalized approaches used to reflect individual needs?	Prevents reproduction of structural inequities; supports equity and participation.
4. Expanding NLP- and functioning-related research	• Does the model capture domains beyond clinical function (e.g., participation, environment, fatigue)? • Are underrepresented or evolving concepts (e.g., accessibility barriers, assistive technologies) included? • Are annotation frameworks co-developed with PwDs?	Enhances recognition of social participation and environmental constraints.
5. Interpretability and clinical usability	• Are model outputs transparent and understandable to PwDs and practitioners? • Are privacy, consent, and user autonomy respected?	Supports autonomy and relational dignity; enables contestability of AI outputs.

While grounded in this synthesis, the study has limitations. As a narrative review, it is subject to selection bias and interpretive judgment, and the evidence base remains heterogeneous. Given the rapid evolution of AI research, some recent developments may not be fully captured.

## 9. Conclusions

This review demonstrates that current AI/ML applications in disability and health only partially reflect justice-oriented frameworks. While these technologies do offer important opportunities for improving early intervention, personalized care, resource allocation, and accessibility, most models remain grounded in biomedical and functional understandings of disability. However, disability like many chronic health conditions is inherently multidimensional, encompassing not only clinical characteristics but also social, environmental, and structural determinants. As a result, they insufficiently capture broader dimensions of wellbeing, including capabilities, recognition, and structural inequities.

By applying disability justice frameworks as an analytical lens, this study highlights a persistent gap between theoretical commitments to equity and their empirical operationalization in AI/ML systems. Current approaches often fail to adequately incorporate social context, lived experience, and systemic barriers, as well as the interaction between clinical and non-clinical factors, limiting their ability to address the complex determinants of health inequities among persons with disabilities.

Bridging this gap requires not only technical innovation but also a fundamental shift toward justice-oriented design and evaluation. This includes the development of disability-inclusive datasets, participatory co-design with persons with disabilities, and the integration of fairness, transparency, and accountability throughout the AI/ML lifecycle. Future approaches must move beyond siloed clinical models to integrate clinical, social, and structural dimensions in a holistic and intersectional manner. Advancing equitable AI in disability and health will depend on interdisciplinary collaboration and sustained attention to both ethical and structural dimensions of technology development. Aligning AI/ML systems with justice-oriented frameworks offers an opportunity to move beyond narrow biomedical models toward more inclusive, context-sensitive, and equitable health systems.
